# A comparison of time to event analysis methods, using weight status and breast cancer as a case study

**DOI:** 10.1038/s41598-021-92944-z

**Published:** 2021-07-07

**Authors:** Georgios Aivaliotis, Jan Palczewski, Rebecca Atkinson, Janet E. Cade, Michelle A. Morris

**Affiliations:** 1 School of Mathematics, University of Leeds, Leeds, LS2 9JT UK; 2 Leeds Institute for Data Analytics, University of Leeds, Leeds, LS2 9JT UK; 3 Alan Turing Institute, British Library, London, NW1 2DB UK; 4 Nutritional Epidemiology Group, School of Food Sciences and Nutrition, University of Leeds, Leeds, LS2 9JT UK; 5 School of Medicine, University of Leeds, Leeds, UK

**Keywords:** Cancer, Breast cancer, Cancer epidemiology, Epidemiology

## Abstract

Survival analysis with cohort study data has been traditionally performed using Cox proportional hazards models. Random survival forests (RSFs), a machine learning method, now present an alternative method. Using the UK Women’s Cohort Study (n = 34,493) we evaluate two methods: a Cox model and an RSF, to investigate the association between Body Mass Index and time to breast cancer incidence. Robustness of the models were assessed by cross validation and bootstraping. Histograms of bootstrap coefficients are reported. C-Indices and Integrated Brier Scores are reported for all models. In post-menopausal women, the Cox model Hazard Ratios (HR) for Overweight (OW) and Obese (O) were 1.25 (1.04, 1.51) and 1.28 (0.98, 1.68) respectively and the RSF Odds Ratios (OR) with partial dependence on menopause for OW and O were 1.34 (1.31, 1.70) and 1.45 (1.42, 1.48). HR are non-significant results. Only the RSF appears confident about the effect of weight status on time to event. Bootstrapping demonstrated Cox model coefficients can vary significantly, weakening interpretation potential. An RSF was used to produce partial dependence plots (PDPs) showing OW and O weight status increase the probability of breast cancer incidence in post-menopausal women. All models have relatively low C-Index and high Integrated Brier Score. The RSF overfits the data. In our study, RSF can identify complex non-proportional hazard type patterns in the data, and allow more complicated relationships to be investigated using PDPs, but it overfits limiting extrapolation of results to new instances. Moreover, it is less easily interpreted than Cox models. The value of survival analysis remains paramount and therefore machine learning techniques like RSF should be considered as another method for analysis.

## Introduction

As machine learning methods continue to evolve and challenge the use of classical statistical approaches used by epidemiologists there will be unavoidable debates about how the two approaches compare, their suitability and whether machine learning techniques conflict with or complement classical statistics. In this article we examine two approaches and discuss how they compare in the context of survival analysis, the backbone of many epidemiological studies. We focus on time until breast cancer incidence using data from UK Women’s Cohort study (UKWCS), a large cohort set up to investigate the links between diet and chronic disease in the 1990s^1^. Breast cancer has been the subject of many epidemiological studies reported in the literature and the effects of factors like obesity (which we focus on here) are well known. This serves the purpose of this study well, which is not to extract epidemiological conclusions but to compare the two approaches: (generalised) linear statistical modelling and fully non-linear machine learning.

The most commonly used linear model in survival analysis is the Cox Proportional Hazards model. Cox Proportional Hazards assumption ($h\left(t\right)={h}_{o}\left(t\right)\mathrm{exp}(\sum_{i}{\beta }_{i}{X}_{i})$, where $h(t)$ is the hazard ratio at time $t$,${\beta }_{i}$’s are coefficients and ${X}_{i}$’s are covariates.) means that there is a linear relationship between the predictors and the log hazard function at any time ($\mathrm{log}\left(h\left(t\right)\right)=\mathrm{log}\left({h}_{o}\left(t\right)\right)+\sum_{i}{\beta }_{i}{X}_{i}$)^2^. Cox models have been used widely to investigate the impact of lifestyle factors and personal characteristics, including physical activity^3^, obesity^4^ and ethnicity^5^ on incidence of breast cancer or other chronic disease. Such models have previously been applied to the UK Women’s Cohort Study (UKWCS) to investigate a range of dietary characteristics (see for example: meat consumption^6^, fibre intake^7^, dietary pattern^8^) and Body Mass Index (BMI)^9^, in relation to breast cancer incidence. Interpretation of these classical models is mainstream in epidemiology. Flexible extensions of the classical Cox Proportional Hazards model are available in the literature^10^ and in statistical package functionality. However, here we focus on the classical Cox Proportional Hazards model as it has been used in the extant literature for UKWCS.

Note that we are investigating time to breast cancer incidence and that censored observations are indeed taking place randomly and independently of the event of interest. Therefore, there is no need to adjust the survival curves estimates for competing risks. We do however use age adjusted Cox regression as participants joined the UKWCS at different ages.

Recently the potential for the use of random forests, along with other machine learning techniques, in epidemiological research has been identified, but are yet to be well understood as a common epidemiological tool.

Weng et al.^11,12^ test a range of machine learning techniques, including random forest, on anonymized electronic medical records from nearly 700 UK family practices to predict cardiovascular disease episodes and find an increased accuracy in the predictions compared to classical statistical models. These methods have been applied in a number of genetic epidemiology genome wide association studies, where data are abundant and complex^12–15^.

Random forests are a collection of decision trees each ‘grown’ on a bootstrap sample of a data set^16^. Starting with the whole sample the tree splits (branches out) the data into nodes using splitting rules (based on randomly selected subset of variables) which maximise the difference between outcomes in each child node. Final nodes are the leaves of the tree.

Random survival forests (RSFs) are an extension of the approach used for right-censored survival data. They have a splitting rule which maximises the difference in survival between child nodes, or leaves^17,18^. Each leaf has a survival function determined by the censor and/or death times of the members of that leaf. The forest predicts a survival function for an individual by averaging the survival functions belonging to each of the leaves they fall into when dropped down the trees. These methods make no assumptions about what the form of the association between variables and outcome is, however, they are often seen as black box models and can be difficult to interpret.

In some cases, the proportional hazards assumption made by Cox models is incorrect, but these cases are not necessarily easy to identify. Statistical tests developed are based on Schoenfeld residuals^19,20^. For example, it is known from previous research^21^ that the hazard due to weight status differs in pre- and post-menopausal women. As menopause is related to age this implies that the proportional hazards assumption for the whole age range is also violated. The complex relationships between variables require unpacking using methods aimed at reducing bias such as Directed Acyclic Graphs^22^ and by running additional models on subsets of the data when an interaction is suspected (for example separate models are often run for pre- and post-menopausal women).

An RSF model imposes no constraints on the relationship between variables and as such may spot this relationship in menopause status without running a model specifically searching for it. However, RSFs perform poorly at extrapolation or interpolation in variable space where the training data are sparse. Although interpretation of RSF models is difficult, exploring the complex relationships between variables can be facilitated by the use of Partial Dependence Plots (PDPs)^23^. PDPs are an approach to visualise relationships between variables, and are useful for knowledge discovery, for example: investigating systolic heart failure patients^24^. We validate model performance using Harrell’s c-index (C)^25^ for discrimination and Integrated Brier Score (IBS) for calibration.

In this paper, we compare a random survival forest model to the classical Cox model when used to investigate the effects of weight status and other factors on the time to breast cancer incidence in the UK Women’s Cohort Study. The robustness of the Cox coefficients is tested using bootstraping and the advantages and drawbacks of each model are highlighted and their predictions are compared.

## Methods

### Data

Analysis was carried out on the UK Women’s Cohort study (UKWCS), a large cohort set up to investigate association between diet and chronic disease in the 1990s^1^. At baseline 35,372 women were recruited and completed postal questionnaires. Ethical approval was obtained from 174 local ethics committees during 1994 and 1995^26^.

The women were followed up with cancer incidence and mortality reports through NHS Digital. Breast cancer incidence data were unavailable for 879 women due to inability to match on NHS number, so analysis was carried out on the remaining 34,493 women. Of these women 1571 (4%) developed breast cancer in a mean time to follow up of 15.3 years. Breast cancer incidence was defined as women free of cancer (except non-melanoma skin cancer) on the date of questionnaire completion at the beginning of the study (1995) who developed malignant breast cancer coded using ICD9 or 10 codes, before the end of the censor date (specific date 1st April 2014).

Women self-reported their height and weight at the time of the questionnaire which were used to calculate Body Mass Index (BMI) and a World Health Organisation defined category was assigned as follows; underweight for BMI less than 18.5; normal weight (N) between 18.5 and 24.9; overweight (OW) between 25 and 30 and obese (O) over 30. They also answered questions about diet using a Food Frequency Questionnaire and alcohol consumption at baseline, following which, nutrient intake values and ethanol consumption in grams per day was calculated.

Time to event is defined at the time the women entered the cohort until the censor date or incidence of breast cancer.

Variables used are known to be risk factors of breast cancer in the literature^29–32^. RSF and Cox models were built using age, height, hormone replacement therapy status, alcohol consumption and folate intake at the time of completing the questionnaire, the number of times the women reported being pregnant, the computed total walking time calculated from reported walking time in summer and winter and dietary pattern. Dietary pattern is a previously defined variable created using cluster analysis on the initial food frequency questionnaire of the UKWCS^33^.

We fitted 3 different Cox models, one on all data with menopause status as a covariate, one only for women who joined the study postmenopausal and one for all data including interactions between menopausal status and weight status.

### RSF and exploring variable relationships

Random survival forests were grown using the “randomForestSRC” package in R^34^. Random forests like other machine learning techniques, often learn better on balanced data^35^, though this is not a requirement. To ensure a level playing field between RSFs and Cox models breast cancer cases were not up-sampled in the models reported. The possibility of up-sampling breast cancer cases using weighted samples was also explored (not reported here) with minor differences in the results (mainly dampening slightly the overfitting of RSF). The random survival forests internal data imputation procedure was used for missing data.

PDPs^23^ were used to investigate the effect of a single variable on the predicted time to event generated by the forest. The forest makes predictions of time to breast cancer incidence for each individual set of variables, but the impact of changing a single variable an overall random forest prediction, is not clear. In order to investigate the average impact of one variable, the whole dataset is modified to take the same value for the variable of interest and breast cancer at 15 years is predicted. For continuous variables this is repeated for a range of values and for categorical variables prediction is made for all possible values. PDPs show the average of these predictions over the whole dataset. To investigate interactions between variables, PDPs will be plotted with additional partial dependence (PD) on a second variable by setting that to a fixed value as well. Confidence intervals for RSF models were calculated using standard errors in the mean of the predicted breast cancer event as suggested by Ishwaran and Kogalur^34^ but should be interpreted with caution.

### CoxPH and bootstrapping

**Figure 1:**
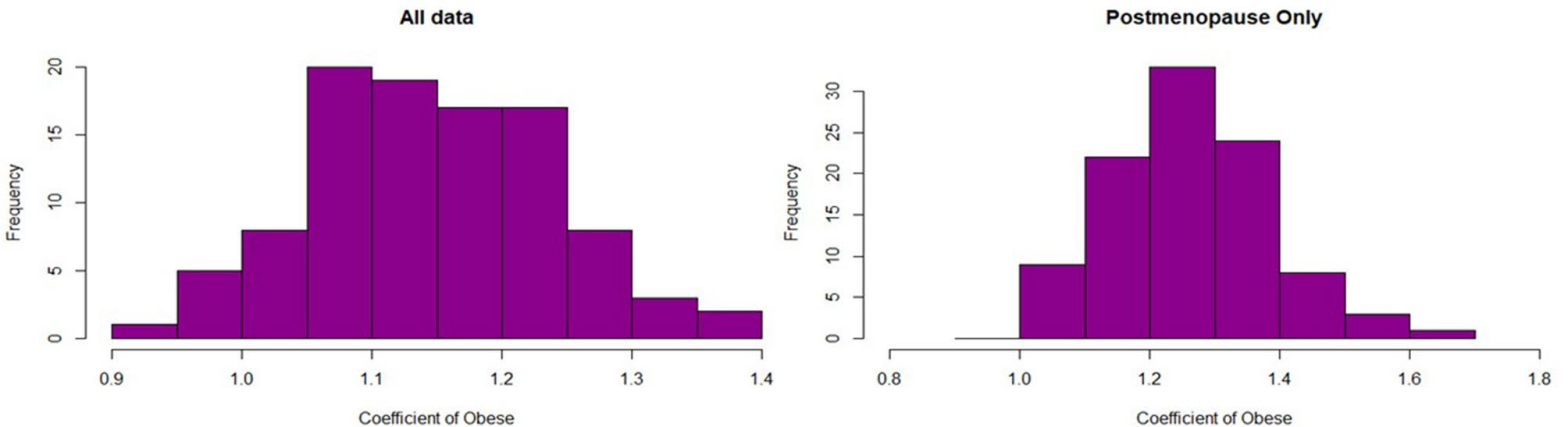
Histograms of coefficients of variable O (Obese) for Cox models trained on 100 bootstrap samples, illustrating that the distribution of the coefficients agrees with the reported confidence intervals for HRs.

Cox models were used to estimate hazard ratios (HR) and 95% confidence intervals (CI) using the “survival” and “pec” packages in R^36-38^. 13,463 (39%) of the records in UKWCS dataset had missing data in one or more of the variables (most missing values are related to the number of pregnancies due to no pregnancies often being recorded as missing values). Multiple imputation was performed using the “mice” package in R^39^. Cox models were trained on the whole dataset (regardless menopause status) and post-menopause only women. In order to check consistency of the HRs, we run Cox models on 100 bootstrapped data samples. Each bootstrap sample was taken with replacement and was equal to the size of the training dataset. Imputation took place after the bootstrap sample was selected. Cox models were built on each bootstrap sample and histograms were created (see Fig. 1) to show the distribution of the coefficients for each model built on bootstrap sample. The distribution of the coefficients is indicative of whether the results of the full dataset are robust to small changes in the composition of the sample.

### Comparison between RSF and CoxPH

Odds ratios (ORs) for the odds of breast cancer incidence before 15 years were generated for RSF to provide comparison with the Cox HRs and to summarise the effect of that variable over this time period. To generate the odds ratio from the RSF the probability of incidence can be found from the survival function: $P(incidence\,at\,t\le T|X_{1}=b)=1-S(T|X_{1}=b)$, where $S(T|{X}_{1}=b)$ is the survival function generated by a PDP with partial dependence on variable ${X}_{1}$ having a constant value $b$. From this probability, odds ratios were generated.

In traditional models such as logistic regression and Cox models the ORs or HRs are fixed values which fully specify the model. In random survival forest models the model is specified only by the splits in every tree in the forest and ORs are estimated from the predicted survival generated by the model. ORs generated by RSFs do not have to be stable for changes of other variables, for example menopause status.

ORs and HRs are not equivalent. HRs refer to hazard, the likelihood of an incidence at a given instance in time whereas ORs refer to the ratio of the probability that an incidence will occur before a given time compared to this not happening. In order to convert ORs to HRs, the baseline hazard or survival would need to be estimated.

Estimating the non-parametric baseline hazard or some parametric form is possible and indeed useful if one wants to compare the effect of a variable as captured by different models. Here, however, we propose the following theorem that allows us to compare the form of the models produced (positive or inverse relationships) and the relative magnitude of the ratios.

#### Theorem 1

*An HR of 1 is equivalent to an OR of 1 and an HR greater (smaller) than 1 is equivalent to an OR greater (smaller) than 1.*

(See Appendix B for the proof).

### Evaluation

Significance in traditional models is checked using a confidence interval and quantified by the p value which tells us the probability of obtaining the results if the null hypothesis, that all HR = 1, were true. To calculate variable importance for an RSF, first the prediction error is calculated for the case where the data set is dropped down all trees of the forest but at each node for the variable of interest the daughter node is chosen randomly. Variable importance is then defined as the difference in prediction error between this case and the actual prediction error^16,17^.

Both Cox and RSF models were K-fold cross validated (K = 100). Harrell’s c-index^25^ is a measure of the predictive power of a survival model and was computed for both RSF and Cox proportional hazards. We additionally report Integrated Brier Score (IBS) for all models. Several other measures of performance for survival models are available and readily produced by R packages, e.g. Nagelkerke, ${R}^{2}$, slope shrinkage, discrimination index, the unreliability index and more. IBS and Harrell’s C-index are well understood and established measures both for Cox models as well as RSF, so we favour these for our research. C-Index considers every possible pair of outcomes that are observed and measures the proportion of cases for which the model predicts correctly which has the better outcome (longer time to breast cancer incidence)^17,25,28^. It is therefore assessing how well the model ranks instances based on their risk. IBS on the other hand, is testing for accuracy of predicted probabilities directly by comparing them to the status at selected times, i.e. measures calibration of the model to the date. The two measures evaluate different aspects of model accuracy. We compare c-indices between models using box plots.

All analysis was carried out in R version 3.6.3. The R script is available at: https://github.com/matga-leeds/RSF_v_Cox_on_UKWCS/blob/1656d93780e96d98f3336fbeb71798449a335faf/code_public.R.

## Results

Initially, it was confirmed (through Kaplan–Meier curves and a chi-squared test) that the proportional hazards assumption held. Table 1 summarises the results from Cox analysis and from an RSF of the data set with both pre- and post-menopause women and the data set for post-menopause women only, with normal weight used as reference category. All coefficients of other model covariates are not reported as these variables were used to adjust for confounders. Confidence intervals are given for the coefficients in order to assess significance. We see that the distributions from the bootstrap samples largely support the conclusions on significance from the confidence intervals.

**Table 1:** Summary of Cox and RSF models reporting HRs and ORs for breast cancer incidence by weight status, when compared to normal weight status.

	Cox proportional hazards		RSF	
	HR (95% CI)	C-Index/IBS	OR (95% CI)	C-Index/IBS
**All**				
UW	0.84 (0.51, 1.36)	0.57/0.025	1.10 (1.08, 1.12)	0.53/0.021
OW	1.13 (0.98, 2.30)		1.24 (1.21, 1.27)	
O	1.16 (0.94, 1.42)		1.36 (1.33, 1.39)	
**Post-menopause**				
UW	0.55 (0.23, 1.34)	0.56/0.028	1.11 (1.09, 1.13)	
OW	1.25 (1.04, 1.51)		1.34 (1.31, 1.70)	
O	1.28 (0.98, 1.68)		1.45 (1.42, 1.48)	
**All with interaction**				
UW	0.50 (0.21, 1.22)	0.57/0.025		
OW	1.22 (1.02, 1.47)			
O	1.24 (0.96, 1.60)			

RSF predicts significantly higher OR for overweight post-menopausal women relative to all women. Since there are more post-menopausal women in the sample the coefficient for the dataset with post- and pre-menopause women (that is regardless of menopause status) is still significant. This was the same for the model with up-sampling, not reported here. This is not true for Cox PH because the inverse effects of being overweight between pre- and post- menopausal status inflate the estimation error. See the histograms of Fig. 1 for the coefficients of the OW variable in the 100 bootstrap sample-based Cox PH models. Note that the histograms of the coefficients are in line with the results reported in Table 1. The reason we include them in this study is to point out the potential variability of the coefficients when there are small changes in the sample.

**Figure 2:**
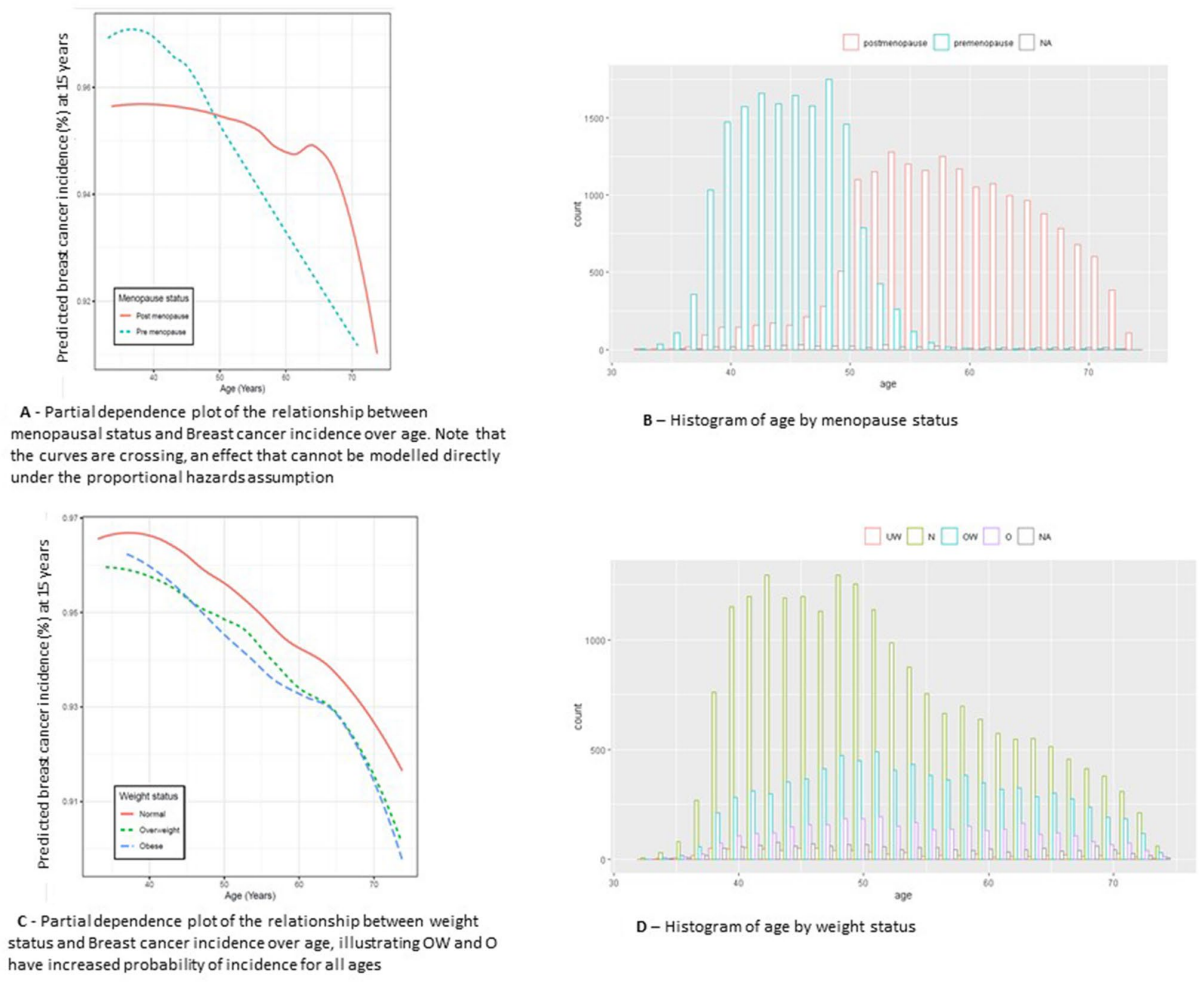
(**A**) Partial dependence plot of the relationship between menopausal status and Breast cancer incidence over age. Note that the curves are crossing, an effect that cannot be modelled directly under the proportional hazards assumption; (**B**) Histogram of age by menopause status; (**C**) Partial dependence plot of the relationship between weight status and Breast cancer incidence over age, illustrating OW and O have increased probability of incidence for all ages; (**D**) Histogram of age by weight status.

The age PDP in Fig. 2A shows a different picture for time to event for pre- and post-menopausal women. The probability of non-incidence within 15 years for pre-menopausal women starts higher than that of post-menopausal ones but reduced much faster with age. Although this is partly due to there being less pre-menopausal than post-menopausal women in the data, a Cox model cannot spot this change in the age variable as it forces a constant HR for all ages. These plots show additional freedom to that available in a Cox model as the PDP plots for Cox models are constrained to be monotonically increasing for HR greater than one or decreasing for HR less than one. For the equivalent notion of a PDP in a Cox model see Appendix B.

Weight status in relation to time to breast cancer incidence in pre-menopausal women tends to show an inverse association (overweight and obese appearing to be protective) and in post-menopausal women a positive association (increasing risk with increasing weight). Although this relationship influences the ORs and HRs (see comment in results above) this is not reflected in the PDP of Fig. 2C where lines move in parallel.

The Cox model assumes a relationship between the variables that obey the proportional hazards assumption. It is expected that the effect of the interaction between weight status and menopause status cannot be represented within this assumption because previous research has found inverse relationships between weight status and breast cancer pre-menopause and positive relationships post-menopause^40^. Including these interactions explicitly in the model results into shifting the non-interaction coefficients closer to the ones from the post-menopause only model. The interaction coefficients are not significant indicated by the inflated variance as explained above and only the coefficient for OW is significant.

The RSF allows interaction between variables to account for this relationship so odds ratios were generated from the existing RSF model with additional partial dependence on menopause. If we did not expect this interaction to have an effect there would be no way to easily identify it with a Cox model, however it could be easily identified in the RSF using PDPs with PD on weight status and menopause.

Post-menopause HR increased with increasing weight status OW 1.25 (1.04, 1.51) and O 1.28 (0.98, 1.68).This is a commonly reported trend^40^. Bootstrap sample models confirm the above results. In the whole data (regardless of menopause status), we see that the histograms of the coefficients go below 1 whereas most values for post-menopause only data stay above 1 (see Fig. 1). For the random survival forest, the first column shows ORs predicted with partial dependence on menopause status only.

**Figure 3:**
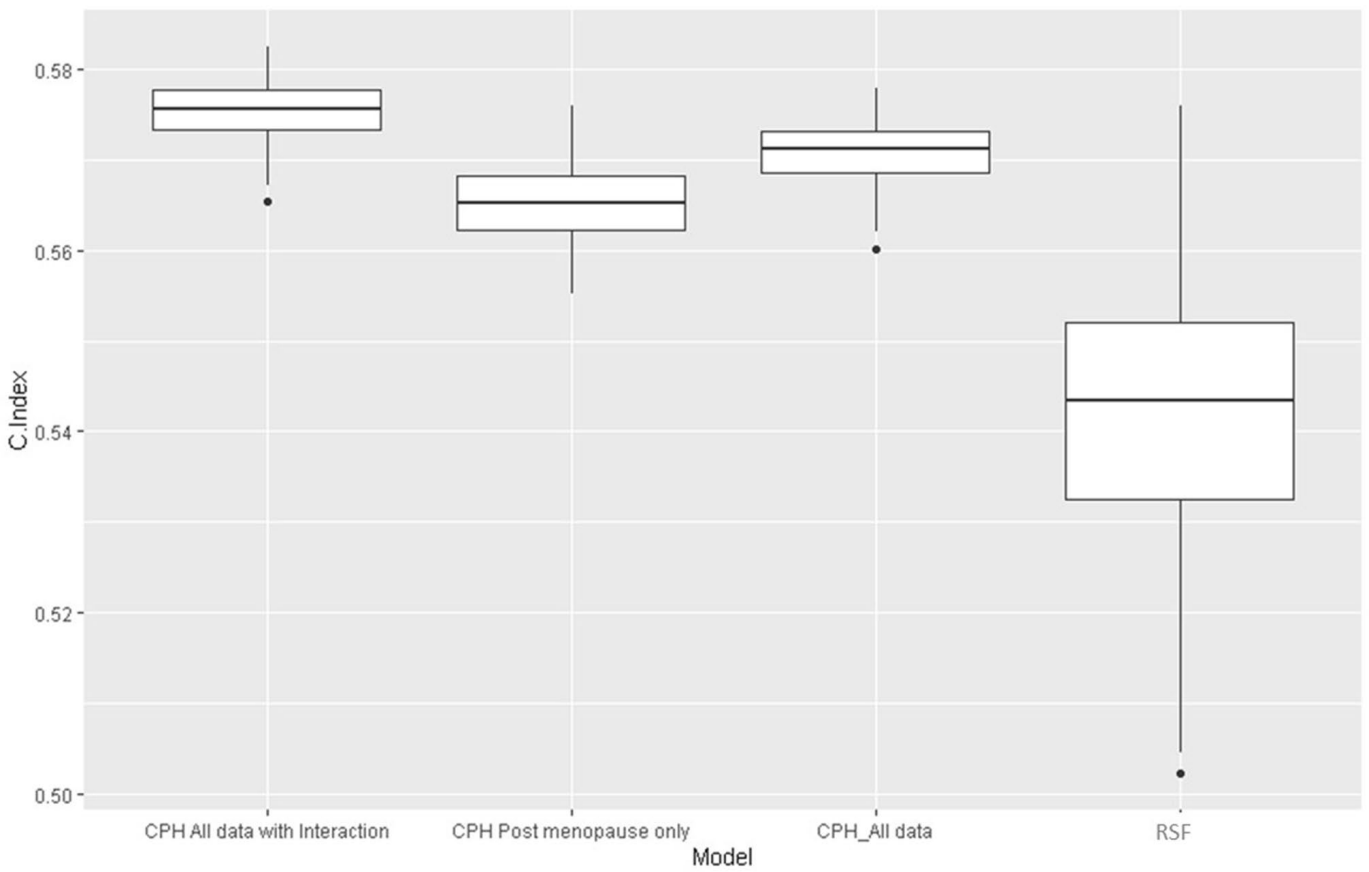
Boxplot showing the c-indices for three Cox Proportional Hazard Models (all data with an interaction term, Post-menopausal women only, the full sample) and the Random Survival Forest.

The random survival forest has very high training sample c-index (approximately 95%) but testing sample c-index of only 0.53. This suggests that the model fit by the random forest overfits the data by learning closely specific instances, hence the variables used in the forest do not describe much of the variance in the outcome. The Cox models have a testing sample c-index of 0.57 and 0.56 respectively, so have predictive power for new data better than that of the RSF which additionally shows wider variability. It is clear that all models have poor predictive ability (0.5 would indicate random guessing) in terms of ranking women based on risk. This is not surprising in datasets where risk is low and the vast majority of women are incidence-free at censoring time. See the box plot in Fig. 3 for comparison.

Both Cox and RSF models however appear to have very high Integrated Brier Score (IBS) in the area of 0.025–0.028 for Cox models and 0.021 for the RSF respectively. This is due to the relative low number of breast cancer cases in the sample making prediction, rather than ranking (see C-Index) an easier task. Note that only 4% of the data are actual instances of breast cancer at 15 years.

## Discussion

Both Cox Proportional Hazards and RSFs confirmed significant increased risk of breast cancer incidence for increased weight status post-menopause. In order to get the best out of both methods, however, it is important to approach the methods with caution and to keep in mind their strengths and weaknesses.

### Advantages and disadvantages of Cox proportional hazard models

In this paper we have shown that Cox models are effective at identifying the relationship between breast cancer and its covariates, at least for the dataset examined, but the process of investigating interactions relies heavily on knowledge of previous research or intuition and a priori causal planning. In cases like the one we investigated above it requires additional models to be run (such as pre- and post-menopause). It may be sensible to calculate a test for trend on the weight variable and possibly use it as a continuous predictor. Furthermore, here we were mainly comparing the two approaches and not investigating the epidemiological problem in depth. What is more, it is unlikely that using the continuous variable would clarify things because firstly, some categories (underweight) have very few data and secondly, due of the non-linear relationship between weight and menopause.

### Interpreting Cox proportional hazard models

The Cox model is sensitive to perturbations in the sample consistency. In big data situations, additional care is needed when applying traditional models. In cases where there are many variables, multiple combinations of these variables could be used for model adjustment. Therefore, it is likely that in some of these combinations a high significance value is found (a multiple testing phenomenon). Care is needed in such situations to avoid reading too much importance into a single model that may be the only one, of thousands of possible models, that finds a strong relationship^41,42^. This problem can be reduced by running bootstrapped models^43^ and cross validation in assessing fit of models.

### Advantages and disadvantages of RSFs

This paper has demonstrated that RSFs can be used to produce odds ratios for breast cancer incidence and to identify the relationships without the assumptions made by traditional models. RSFs have the advantage that they are non-linear models and so can represent any form of interaction between variables. An RSF can be grown on a data set with a large number of variables, furthermore PDPs can be used to investigate potential interactions between variables. In this way a random survival forest is a more general model and can be used to easily spot new or unexpected trends in the data. Flexible extensions of Cox models can allow for time dependent and even nonlinear effects but are still bound by the dimensionality of the model. These extensions (especially related to time dependent covariates) are not readily available for RSFs. On the other hand, RSFs perform poorly at prediction for data where there was little comparable training data as the lack of model structure makes extrapolation meaningless. Therefore, for extreme values on continuous variables and in categorical variables for which there is little training data (here in the underweight BMI category) predictions are unreliable. By contrast, a traditional Cox model would typically perform better in such cases as it just extrapolates linearly the trends of the model. If random survival forests are interpreted with care and together with PDPs they may give more insight into the associations between risk factors..

However, RSFs as other machine learning methods are very efficient in learning patterns and in datasets with relatively few cases compared to no number of censored observations, like the one examined here, they tend to learn precisely instances and thus overfit.

### Interpreting RSFs

Although random forests deal better with interactions between variables, care is still needed when interpreting PDPs. For example, a forest which uses age, menopause status and weight status is unlikely to produce reliable results in a partial dependence plot for menopause and weight status because relabelling people aged under 40 as post-menopause (erroneously) is creating a set of variables that were likely unprecedented in the training set and as such asking the model to extrapolate the patterns. It is therefore important to be aware when PDP predictions are based upon sufficient training data to be reliable.

RSFs ability to fit any relationship and take any form makes them difficult to summarise. If interactions are largely known and can be accounted for the simplicity of the output HRs that completely define a Cox model is attractive. RSFs in this paper have been used to generate ORs at 15 years follow up. These ORs can be used to draw similar results about relationships as from HRs from the Cox model and so can be used as a summary while the RSF still allows for much more in depth investigation of interactions between variables through PDPs, without the need to introduce new models.

Traditional models where the risk factors describe little of the variance of the outcome tend to have worse training data c-index than RSFs. In this case both models have similar testing c-indices which imply poor predictive power. Poor predictive power is not surprising in these models because breast cancer incidence is relatively low and fairly random in women with any combination of variables but is slightly more prevalent in groups which have certain risk factors.

### Future work

Building on the sparse use of machine learning to date in epidemiology, perhaps in the future, epidemiology will be pursued with the use of both tools and as such our judgment of the value of information produced by each will be more measured. No epidemiologist would claim the whole story of a disease could be explained with a few numbers and yet a lot of significance is read into the hazard and odds ratios produced by models. Interpreting the results of machine learning algorithms is just as fraught, if not more so, with the danger of assigning too much value to the information produced especially at the extremes of the model. In the future pursuit of an accurate understanding of risk factors, RSFs may be used to investigate interactions and then traditional models used to summarise the results. This may be achieved by training RSFs on large datasets and then using a series of PDPs to identify any interactions that may influence results. A Cox model may then be trained on a subset of data that avoids interactions to produce HRs that summarise the risk. Further work is required to unpack why these RSF models overfit.

## Conclusion

Post-menopausally, increased BMI has been found to be a risk factor for breast cancer in the UKWCS. This has been achieved using traditional Cox models and using an RSF. The structure of the RSF model is not as easy to interpret as a Cox model and overfits, limiting extrapolation of results to new instances. Generating ORs for a time towards the end of the study period helps to summarise RSF models and compare them with Cox models. RSF can be used to investigate in more detail the interactions between variables and allows forms of interaction to be interpreted that are prevented from being observed by the assumptions of the Cox model. Caution is needed when interpreting the results of either model to ensure an appropriate amount of importance is read into their results. Both approaches have merit and could be used in combination to provide further insights.

The Cox Proportional Hazard method still has high utility in epidemiological research but this paper shows that RSFs could be considered as an alternative or complementary method.

## Supplementary Information

Supplementary Information.
