# Supplementary material for: A comparison of time to event analysis methods, using weight status and breast cancer as a case study
Source: Sci Rep. 2021 Jul 7;11:14058. doi: 10.1038/s41598-021-92944-z (PMC8263588; doi:10.1038/s41598-021-92944-z)
Supplement: Supplementary file 1 — Supplementary Information. [file 41598_2021_92944_MOESM1_ESM.docx]

**A comparison of time to event analysis methods, using weight status and breast cancer as a case study**

Georgios Aivaliotis^1,2,3^, Jan Palczewski^1,2^ , Rebecca Atkinson^2^, Janet E. Cade4and Michelle A Morris^2,3,4,*^

School of Mathematics, University of Leeds, LS2 9JT

^2^ Leeds Institute for Data Analytics, University of Leeds, LS2 9JT

^3^ Alan Turing Institute, British Library, London, NW1 2DB.

^4^ Nutritional Epidemiology Group, School of Food Sciences and Nutrition, University of Leeds, LS2 9JT

^5^ School of Medicine, University of Leeds.

*Corresponding author: M.Morris@leeds.ac.uk

All authors made equal contribution.

# APPENDIX A: TERMINOLOGY

OR – Odds Ratio - a ratio of the odds of an event happening, here the event is breast cancer incidence. For RSF the OR will be evaluated at a time to give odds ratio of an event occurring before a given time.

HR – Hazard Ratio – a ratio of the hazard (probability of an event at an instant in time) of an event, here the event is breast cancer incidence.

Bootstrap – A sample taken with replacement from the initial training data of the same size as the training data.

Survival - Proportion of people that “survive” (or are predicted to “survive”) i.e do not experience an event before a given time, here the event is breast cancer incidence.

**APPENDIX B: SURVIVAL, ODDS AND PARTIAL DEPENDENCE WITHIN THE PROPORTIONAL HAZARDS ASSUMPTION**

**B.1: The Proportional Hazards Assumption**

The proportional hazards assumption is

$$h\left( t \right)=h_{o}\left( t \right)\exp\left( \sum_{i} \beta_{i}X_{i} \right),$$

where $i$ is summed over all variables in the model. It implies that the hazards for any individual at time$t$ is the baseline hazard at $t$ multiplied by the exponential of a linear combination of the variables.

Let individual $j$ be described by the set of variables$X_{i,j}$. Then the hazard of individual j at time t implied by the Cox model is

$$h\left( t,j \right)=h_{o}\left( t \right)\exp\left( \sum_{i} \beta_{i}X_{i,j} \right)$$

For example, with regards to BMI, we have the following (pseudo-) variables

**B.2: Predicted Survival and PDPs**

Survival can be calculated from the cumulative hazard as follows

$$S\left( T \right)=\exp\left( -H(T) \right)=exp\left( -\int_{0}^{T} h\left( t \right)dt \right).$$

Within the proportional hazards assumption predicted survival for individual $j$ is

$$S\left( T,j \right)=exp\left( -exp(\sum_{i} \beta_{i}X_{i,j})\int_{0}^{T} h_{o}\left( t \right)dt \right).$$

Suppose we’re interested in the odds ratio of being weight status overweight (OW) compared to normal (N) which we treat as a reference value in the following discussion, the survival at time $T$ for individual $j$ given they are overweight is given by

$$S\left( T,j|X_{weight status}=OW \right)=exp\left( -exp(\sum_{i\neq weight status} \beta_{i}X_{i,j})exp(\beta_{OW})\int_{0}^{T} h_{o}\left( t \right)dt \right)=\left[ exp\left( -exp(\sum_{i\neq weight status} \beta_{i}X_{i,j})\int_{0}^{T} h_{o}\left( t \right)dt \right) \right]^{exp(\beta_{OW})},$$

We can identify $exp\left( -exp(\sum_{i\neq weight status} \beta_{i}X_{i})\int_{0}^{T} h_{o}\left( t \right)dt \right)=b_{T,j}$ as the reference survival for individual j with variables $X_{i,j}$ and weight status N. Therefore,

$$S\left( T,j|X_{weight status}=OW \right)={b_{T,j}}^{exp(\beta_{OW})}.$$

A partial dependence plot that obeys the proportional hazards assumption would plot the average survival over all individuals $j$ at time $T$ for each weight status. The PDP prediction for OW can therefore be found as

$$\frac{1}{j}\sum_{j} S\left( T,j|X_{weight status}=OW \right)=\frac{1}{j}\sum_{j} {b_{T,j}}^{exp(\beta_{OW})}.$$

**B.3: Comparing HRs and ORs (Proof of Theorem 1)**

Using the notation introduced in the previous sections of the Appendix B, we prove Theorem 1. We produce the proof, without loss of generality, using the variable OW as an example to relate it more to our problem. The results hold for any variable.

Probability of incidence before time $T$ is

$$P\left( incidence | t<T,j,X_{weight status}=OW \right)=1-S\left( T|j,X_{weight status}=OW \right)=1-{b_{T,j}}^{\exp\left( \beta_{OW} \right)}$$

and Odds are

$odds\left( incidence | t<T,j,X_{weight status}=OW \right)=\frac{1-{b_{T,j}}^{exp(\beta_{OW})}}{{b_{T,j}}^{exp(\beta_{OW})}}$.

Therefore odds ratio compared to normal weight status is

$$OR\left( incidence | t<T,j,X_{weight status}=OW \right)=\frac{1-{b_{T,j}}^{exp(\beta_{OW})}}{{b_{T,j}}^{exp(\beta_{OW})}}\cdot\frac{b_{T,j}}{1-b_{T,j}}=\frac{{b_{T,j}}^{-exp(\beta_{OW})}-1}{{b_{T,j}}^{-1}-1}$$

By definition $0<b_{T,j}<1$. Let $c_{T,j}={b_{T,j}}^{-1}$, (then $c_{T,j}>1$) and we have

$$OR\left( incidence | t<T,j,X_{weight status}=OW \right)=\frac{{c_{T,j}}^{exp(\beta_{OW})}-1}{c_{T,j}-1}$$

We note that $exp(\beta_{OW})$ is the $HR$ for OW weight status and so we find the following properties

$$HR=1\Leftrightarrow OR=1$$

$${HR}_{1}>{HR}_{2}\Leftrightarrow\frac{{c_{T,j}}^{\mathrm{HR}_{1}}-1}{c_{T,j}-1}>\frac{{c_{T,j}}^{\mathrm{HR}_{1}}-1}{c_{T,j}-1}\Leftrightarrow{OR}_{1}>{OR}_{2}$$

These properties hold for every individual j and so also hold for the average over all individuals.

**B.4: Properties of PDPs**

It was shown above that predicted survival for individual $j$ within the proportional hazards assumption can be found using

$$S\left( T,X_{i,j} \right)=exp\left( -exp(\sum_{i} \beta_{i}X_{i,j})\int_{0}^{T} h_{o}\left( t \right)dt \right)$$

Suppose we are interested in the effect of the age variable on any individual

$S\left( T,j|X_{age}=x_{age} \right)={exp\left( -exp(\sum_{i\neq age} \beta_{i}X_{i.j})\int_{0}^{T} h_{o}\left( t \right)dt \right)}^{exp(\beta_{age}x_{age})}={b_{T,j}}^{exp(\beta_{age}x_{age})}$.

Recall that $b_{T,j}$ is a constant survival probability so $0<b_{T,j}<1$

Therefore $S\left( T,j|X_{age} \right)$ is decreasing in $X_{age}$ when $\beta_{age}>0$ and increasing when$\beta_{age}<0$. The PDP which plots average survival at time $T$ as a function of age is therefore given by

$$S\left( {T|x}_{age} \right)=\frac{1}{j}\sum_{j} {b_{T,j}}^{exp(\beta_{age}x_{age})},$$

which is also decreasing in $X_{age}$ when $\beta_{age}>0$ and increasing when $\beta_{age}<0$.

A survival curve with 2 partial dependencies on age and weight status in the Cox model can be described by

$$S\left( {T,j|X}_{age}=x_{age},X_{weight}=x_{weight} \right)={b_{T,j}}^{exp(\beta_{age}x_{age})exp(\beta_{weight}x_{weight})}$$

Where $b_{T,j}$ is reference survival for at time $T$ for individual $j$ with variables$X_{i,j}$.

This implies the survival functions for each weight status are related by raising the reference (normal) weight status survival to the power of the HR and therefore the survival functions can never be equal unless they are equal everywhere and the HR is one.

$$S\left( X_{age}|T,j,X_{weight}=OW \right)={b_{T,j}}^{exp(\beta_{age}X_{age})exp(\beta_{OW})}= {S\left( X_{age}|T,j,X_{weight}=N \right)}^{exp(\beta_{OW})}$$

The PDP plot will therefore take the form

$$\sum_{j} S\left( T,j|X_{age}=x_{age},X_{weight}=OW \right)= \sum_{j} {S\left( X_{age}|T,j,X_{weight}=N \right)}^{exp(\beta_{OW})},$$

this implies the PDPs for age with additional partial dependence on weight status cannot intersect as the plot must be less than the reference for $\exp\left( \beta_{OW} \right)>1$ and greater than the reference for $\exp\left( \beta_{OW} \right)>1.$
